# State Board of Health

**Published:** 1875-03

**Authors:** 


### Editorial.

---

The Legislature of Georgia, which has just adjourned, has passed two laws which are of special interest to the medical profession of the State, *i. e.*: First, a law creating a State Board of Health, which Board is charged with the registration of births, deaths and marriages, in addition to its general sanitary duties; and, secondly, a law granting to physicians the same right of garnishment for their fees as has been previously given to grocers and provision dealers for their security for supplies furnished to laborers. The latter law had become a real *necessity* to the community, as well as to the profession throughout the whole State, and will bring hope and joy to the heart of many a lean and lantern-jawed disciple of Æsculapius, whose patrons have unfeelingly driven him to the very verge of starvation.

